# Supplementary material for: Sugar Beverage Habitation Relieves Chronic Stress-Induced Anxiety-like Behavior but Elicits Compulsive Eating Phenotype via vLSGAD2 Neurons
Source: Int J Mol Sci. 2022 Dec 30;24(1):661. doi: 10.3390/ijms24010661 (PMC9820526; doi:10.3390/ijms24010661)
Supplement: Supplementary file 1 [file ijms-24-00661-s001.zip › ijms-2042027-supplementary.pdf]

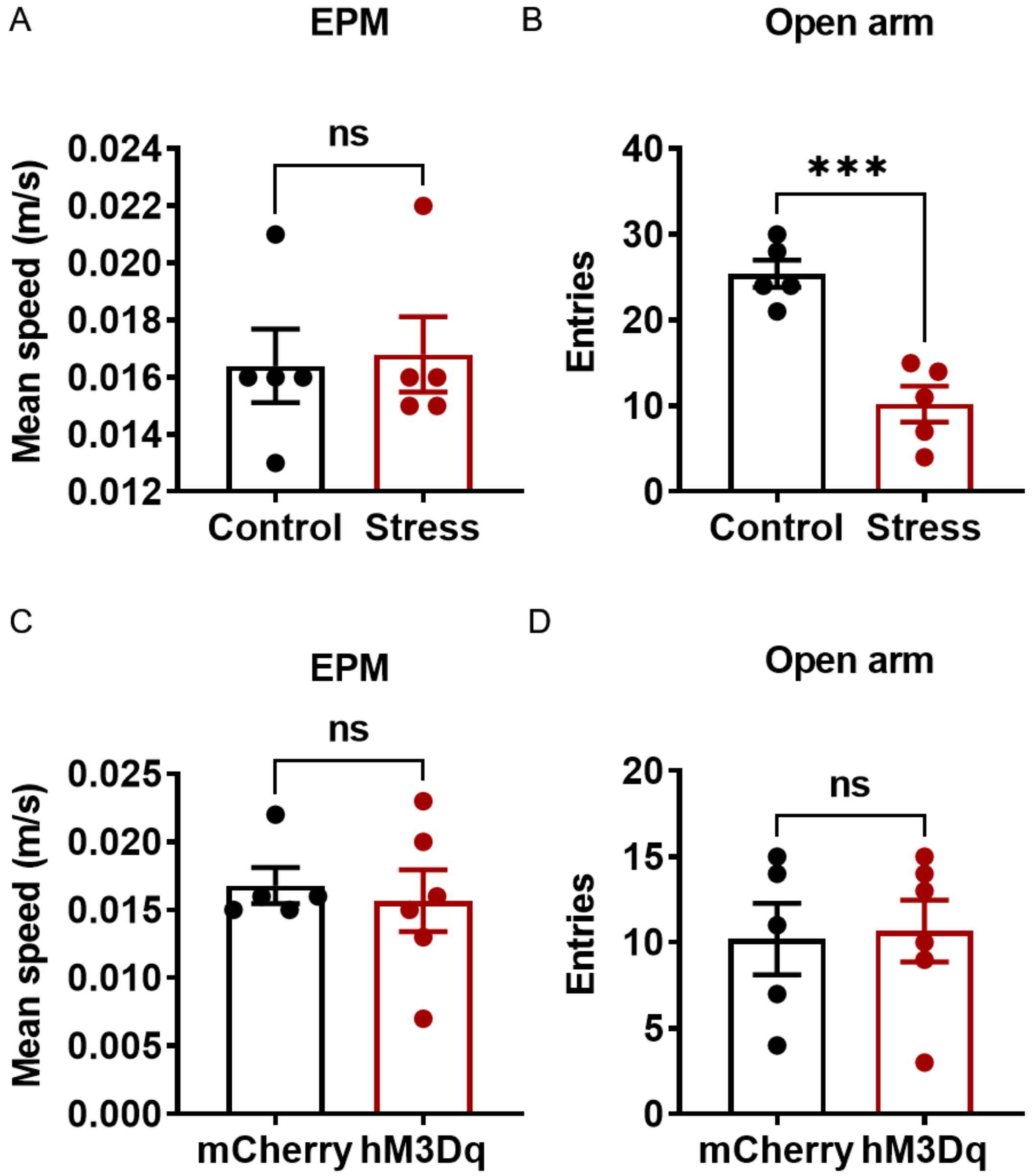

Figure S1: Pharmacogenetic activation of vLS GAD2 neurons did not alter basal anxiety level. (**A**,**B**). Mean speed, number of entries in open arm of EPM after stress. (**C**,**D**). Mean speed, number of entries in open arm of EPM after pharmacogenetic activation vLS GAD2 neurons. N = 5–6. Data were analyzed by student *t*-test and are shown as mean ± SEM. ns, no significant difference. ***, $p < 0.001$.
